# Artesunate enhances the efficacy of enzalutamide in advanced prostate cancer

**DOI:** 10.1016/j.jbc.2025.108458

**Published:** 2025-03-26

**Authors:** Xinyi Wang, Jinghui Liu, Fengyi Mao, Yifan Kong, Qiongsi Zhang, Chaohao Li, Daheng He, Chi Wang, Yanquan Zhang, Ruixin Wang, Sally R. Ellingson, Qiou Wei, Zhiguo Li, Xiaoqi Liu

**Affiliations:** 1 Department of Toxicology and Cancer Biology, University of Kentucky, Lexington, Kentucky, USA; 2 Department of Biostatistics, University of Kentucky, Lexington, Kentucky, USA; 3 Markey Cancer Center, University of Kentucky, Lexington, Kentucky, USA

**Keywords:** artesunate, c-Myc, enzalutamide resistance, prostate cancer

## Abstract

Prostate cancer (PCa) is one of the leading causes of death among men worldwide. Treatments targeting the androgen receptor pathway remain the standard therapy for PCa patients. Enzalutamide (ENZ), a second-generation androgen receptor inhibitor, was developed to treat castration-resistant prostate cancer. However, while patients initially respond to ENZ, drug resistance typically develops within a few months. Artesunate (ART), a semisynthetic derivative of the Artemisinin plant, is approved for antimalaria treatment. In this study, we conducted an FDA-approved drug screening and identified ART as a potential candidate for overcoming ENZ resistance in PCa. Mechanistically, ART induces the degradation of c-Myc, enhancing the efficacy of ENZ. Additionally, patient dataset analysis revealed that c-Myc plays a significant role in developing ENZ resistance. To summarize, these findings suggest a novel therapeutic strategy for ENZ-resistant prostate cancer.

Based on the latest cancer statistics report, prostate cancer (PCa) incidences increase 2 to 3% yearly. Among all diagnosed cancer cases, PCa almost takes up to one-third of all incidences and is the second leading cause of death in men (1). Therefore, it remains a significant challenge for human health. The androgen receptor (AR) plays a key role in PCa initiation and progression. Thus, targeting AR with small molecule inhibitors remains a significant clinical treatment. Regardless of the beginning responses, most of PCa patients develop castration-resistant prostate cancer (CRPC) (2). It is well-established that CRPC is marked by losing sensitivity to androgens yet often exhibits overexpression of AR. As a result, AR still remains an effective target in the CRPC stage. Scientists have invented several second-generation AR inhibitors such as enzalutamide (ENZ), to target AR signaling pathways (3). Although emerging evidence has shown that these AR inhibitors act more effectively in AR activity blockage, PCa patients receiving those inhibitors still develop resistance subsequently (4). Therefore, more investigations are needed to explore novel therapeutics for overcoming enzalutamide resistance (ENZ-R). Although new drug development usually takes a long time to get approved for clinical use, libraries of approved drugs can be screened and provide new insight into combination treatment.

We performed a high throughput screen using the FDA-approved drug library (APEXBIO) and scored a candidate—Artesunate (ART). ART is a semisynthetic derivative from artemisinin plant (5), a traditional Chinese natural medication that has been used for treating malaria worldwide. Several studies have implicated ART’s anticancer potential (6, 7, 8, 9). However, the direct targets of ART remain unclear.

Overexpression of c-Myc is often observed in multiple types of cancer (10). As a master transcription factor, c-Myc regulates diverse networks of cell events, including cell cycle progression, DNA replication, metabolism, and apoptosis (11, 12, 13). Although c-Myc is a favorable target for human cancer, many clinical trials have failed due to low selectivity (14). In this study, the combination of ART and ENZ exhibited strong synergistic effects in both *in vitro* and *in vivo* models. These effects were mediated through the inhibition of c-Myc signaling, epithelial-mesenchymal transition (EMT) suppression, and oxidative phosphorylation disruption, significantly reducing tumor growth and enhancing apoptosis in drug-resistant PCa cells. These findings suggest that ART holds great promise as a potential therapeutic option for overcoming ENZ resistance.

## Results

### Drug screening identifies ART as a therapeutic candidate for ENZ-R PCa cells

**Figure 1:**
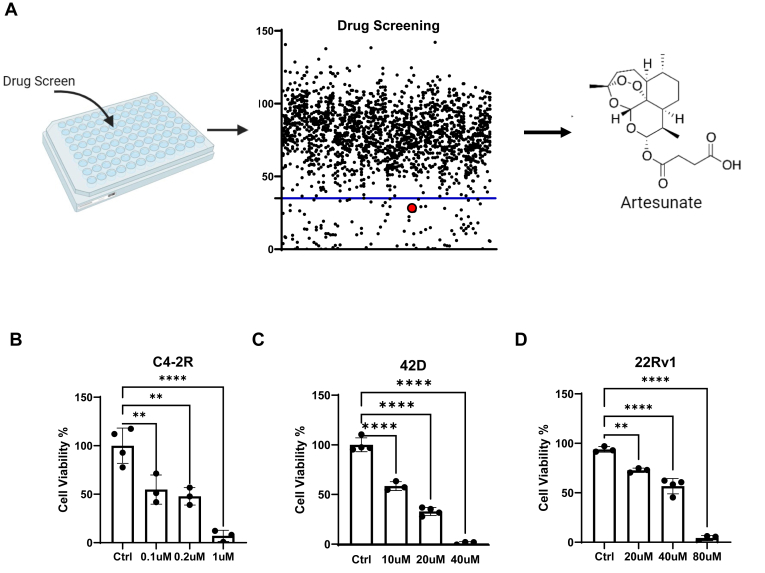
**Drug screening result and validation.***A*, drug screening of 1971 clinically approved compounds from APEXBIO FDA-approved drug library. The *black dot* and *blue line* represent the threshold for positive hit compounds. The *red dot* represents where artesunate is located. *B*–*D*, 3-day cell viability assays to compare different dosages of artesunate inhibiting enzalutamide-resistant prostate cancer cell lines. Data were normalized to groups without any treatment and shown as mean ± SD (with scatter *dot plots* indicating the number of replicates per group). The statistical method used is a two-tailed *t* test. ∗∗, *p* < 0.01. ∗∗∗∗, *p* < 0.0001. These results demonstrate the efficacy of artesunate in inhibiting the growth of enzalutamide-resistant prostate cancer cells.

To identify potential therapeutic agents capable of overcoming ENZ-R in PCa, we performed a high-throughput drug screening using 42D cells developed in Dr Zoubeidi’s Lab (15). 42D cell line is derived from the xenograft of the LNCaP cell line and is specifically selected for resistance to ENZ. We screened the APEXBIO drug library, which is composed of 1971 FDA-approved drugs, at a final concentration of 10 μM. Cell viability was assessed, and we calculated the relative viability of the cells. Among the 1971 compounds in the library, 99 drugs met the threshold of reducing cell viability by 30% or more (Fig. 1*A*). We also conducted a literature review and excluded some candidates that had either been involved in clinical trials or were known to be highly toxic. Ultimately, we identified one candidate: ART, a derivative of the artemisinin plant with established antimalarial properties. We further validated that ART exhibited a dose-dependent reduction in the viability of ENZ-R cells, including the C4-2R, 22RV1, and 42D cell lines (Fig. 1, *B*–*D*). Specifically, ART treatment reduced cell viability by up to 80% at a concentration of 1 μM in C4-2R cells, with similar inhibitory effects observed in the 22RV1 and 42D cell lines at varying concentrations. These results indicate that ART can significantly inhibit the proliferation of ENZ-R PCa cells *in vitro*.

### ART administration downregulates the c-Myc signaling pathway in ENZ-R cells

**Figure 2:**
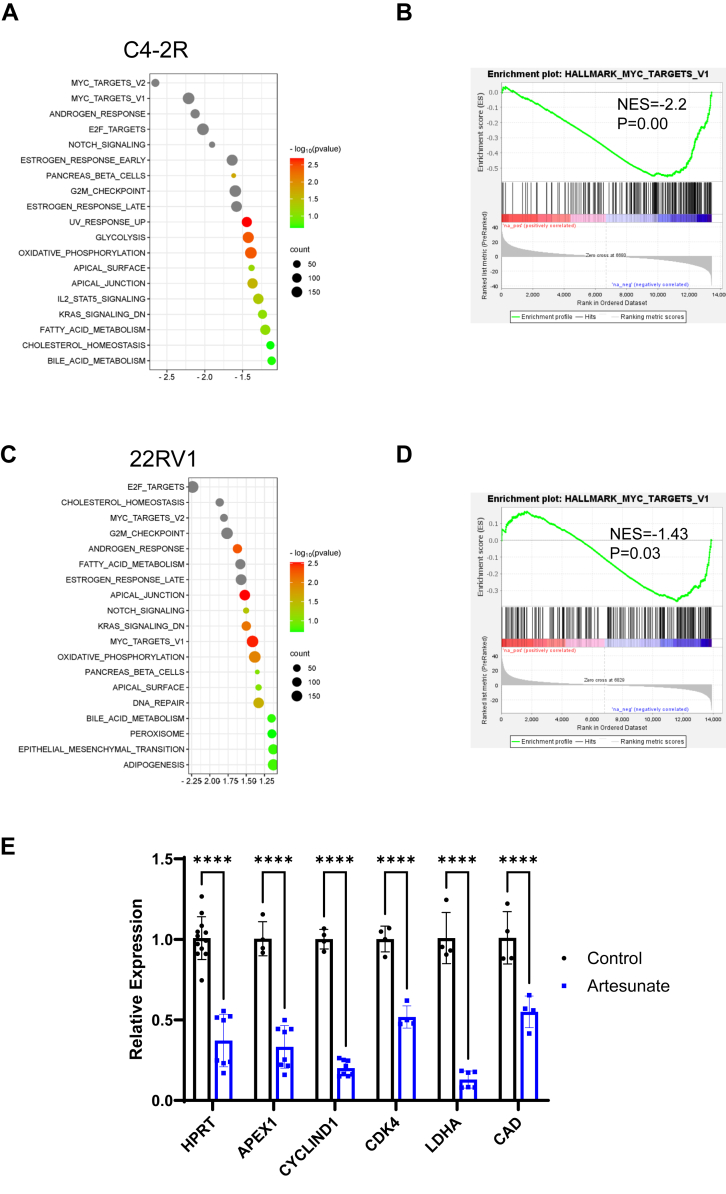
**Artesunate treatment down-regulates c-Myc signaling pathway.***A* and *B*, gene set enrichment analysis (GSEA) to compare pathways related to artesunate inhibition. C4-2R cells were treated with 0.5 μM ART for 12 h and then subjected to RNA-seq analysis. *C* and *D*, GSEA will be used to compare pathways related to artesunate inhibition. 22Rv1 cells were treated with 10 μM ART for 12 h and then subjected to RNA-seq analysis. The GSEA analysis reveals which biological pathways are enriched or depleted following artesunate treatment. *E*, qRT-PCR was used to detect mRNA levels of cMyc and cMyc-targeted genes and demonstrate the impact of artesunate decreases in c-Myc expression and its downstream targets. C4-2R cells were treated ART (0.5 μM) for 12 h with ∗∗∗∗*p* < 0.0001 by the two-tailed, unpaired *t* test.

Since we validated that ART inhibits ENZ-R PCa proliferation, we further investigated the antitumor properties of ART in ENZ-R cells using unbiased RNA-seq analysis in C4-2R cells and 22Rv1 cells. Both cell lines are ENZ-R. C4-2R derived from C4-2 by extensive treatment with a high concentration of ENZ and maintained in 20 μm ENZ culture medium (16). Whereas 22Rv1 was established from CWR22 xenograft, it expresses both AR full length and AR-V7 variant, which lacks a ligand-binding domain and remains continuously active (17). Upon comparing the RNA profiles of cells without ART treatment to the cells with ART treatment, we were able to identify a significant number of genes that are differentially expressed in C4-2R and 22Rv1 cells (Fig. S3). In addition, we used Hallmark predefined pathway analysis, which showed pathways related to PCa progression were significantly downregulated in both C4-2R ART treatment group (Fig. 2*A*) and 22Rv1 treatment group (Fig. 2*C*). Interestingly, in top3 enriched pathways, the c-Myc signaling pathway was significantly suppressed in both C4-2R (Fig. 2*B*) and 22Rv1 cells (Fig. 2*D*). To validate the RNA-seq results, we performed qRT-PCR. As a result, downstream targets of c-Myc, such as cyclinD, CAD, and LDHA, were decreased, consistent with our RNA-seq result (Fig. 2*E*).

### c-Myc is essential for ENZ reponse of ENZ-R PCa patients

**Figure 3:**
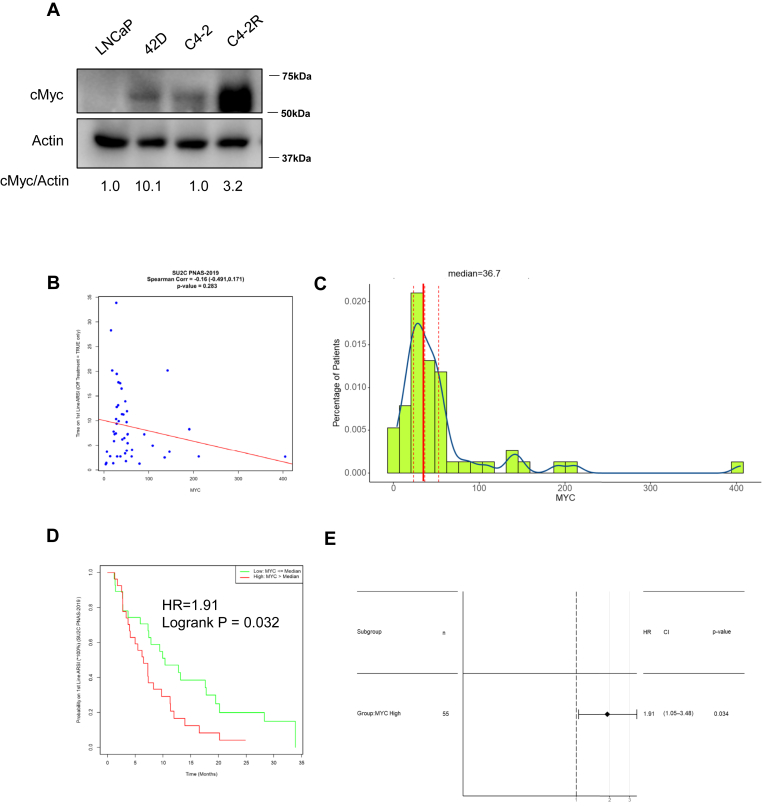
**Association of c-Myc expression with enzalutamide treatment response.***A*, LNCaP, 42D, C4-2, and C4-2R cells were harvested and subjected to Western blot analysis to assess c-Myc expression levels. *B*, the scatterplot uses Spearman’s correlation to evaluate the relationship between the duration of enzalutamide treatment and c-Myc mRNA expression in patients. A linear regression line is included for visualization. The analysis focuses on patients who were labeled as "off treatment" (n = 50), highlighting any potential correlations between treatment duration and c-Myc expression levels. *C*, the histogram illustrates the distribution of FPKM-normalized c-Myc gene levels in a cohort of patients (n = 55). The *dashed* line represents the median expression level, providing a visual representation of the variability in c-Myc expression across the population. *D*, the Kaplan-Meier curve examines the relationship between continued androgen receptor signaling inhibitor (ARSI) treatment and c-Myc expression. Patients were dichotomized into high *versus* low c-Myc expression groups using the median as a cutoff (n = 55). The log-rank *p*-value is included to assess statistical significance. *E*, the forest plot, derived from Cox proportional hazards analysis, compares the efficacy of enzalutamide treatment between patients with high *versus* low c-Myc expression. The results indicate whether the c-Myc-high group experienced more prolonged treatment efficacy compared to the c-Myc-low group. *B*–*E*, results were analyzed from the “SU2C PNAS2019” dataset.

To further confirm the importance of c-Myc in ENZ-R PCa cells, we first examined c-Myc expression levels in ENZ-R cells. We observed elevated c-Myc protein levels in ENZ-R cells (Fig. 3*A*). Our previous publication also mentioned that c-Myc–related pathways were highly upregulated in ENZ-R cells as well (18). As c-Myc is a well-established key driver in cancer and a critical survival factor for cancer cells, we questioned whether c-Myc levels in advanced PCa correlate with the response of patients who received ENZ treatment. Our dataset analysis showed that high c-Myc levels were associated with an earlier failure to respond to ENZ treatment (Fig. 3, *B* and *C*). Further analysis indicated that patients with high c-Myc expression exhibited poor survival rates (Fig. 3*D*). Additionally, a Cox proportional hazards analysis revealed that the c-Myc high group experienced shorter efficacy of ENZ treatment than the c-Myc low group (Supplemental Data 1E). Taken together, these findings suggest that c-Myc could be a promising therapeutic target for overcoming ENZ-R in PCa. Historically, direct targeting of c-Myc in clinical trials has failed due to low specificity and high toxicity (19). However, our observation that ART inhibits c-Myc highlights its potential as a novel therapeutic strategy in ENZ-R PCa.

### ART administration downregulates the AR signaling pathway in ENZ-R cells

**Figure 4:**
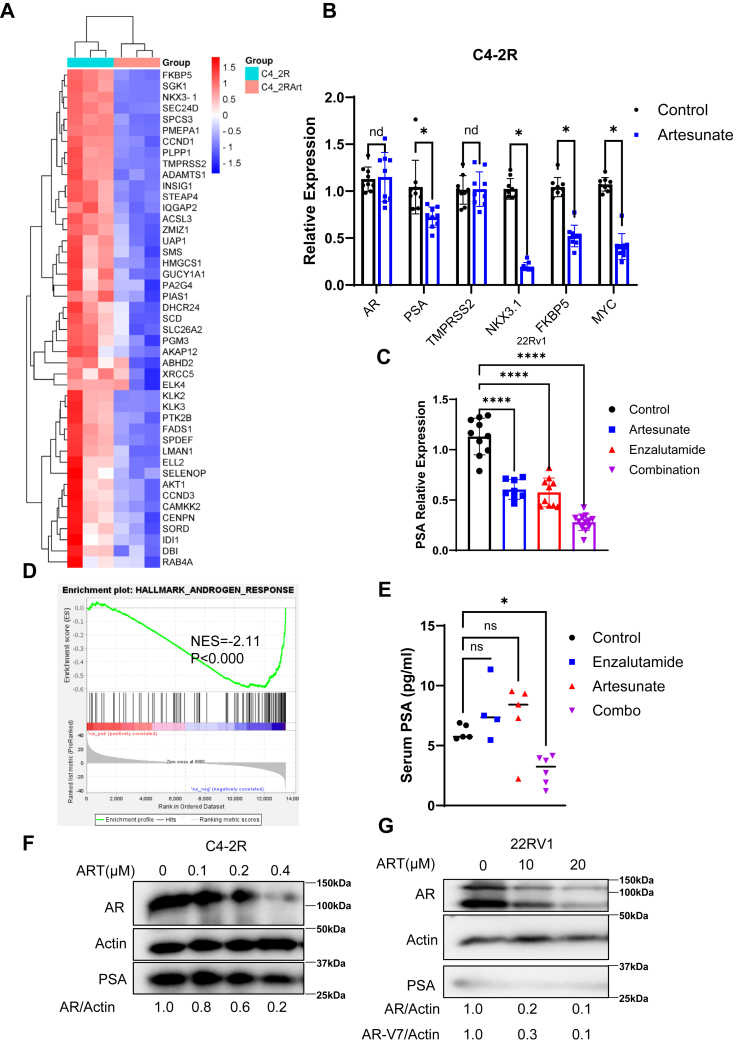
**ART inhibits the AR signaling pathway.***A* and *D*, the results of a heatmap and pathway enrichment analysis using gene set enrichment analysis (GSEA) to examine the androgen signaling pathway. C4-2R cells were treated with 0.5 μM ART for 12 h and then subjected to RNA-seq analysis. The GSEA analysis reveals which biological pathways are enriched or depleted following ART treatment. *B* and *C*, quantitative real-time PCR (qRT-PCR) analysis assessing the mRNA levels of androgen receptor (AR)-targeted genes and demonstrating the effect of ART inhibiting AR-targeted gene expression. Statistical significance was determined using a two-tailed, unpaired *t* test, where ∗ indicates *p* < 0.05, and ∗∗∗∗ indicates *p* < 0.0001. *E*, prostate-specific antigen (PSA) measurements in blood serum harvested from 22Rv1 xenograft mice and evaluates the impact of artesunate treatment, which decreases PSA levels *in vivo*. Data were normalized to the vehicle control group, and statistical significance was determined using a one-way ANOVA test, where ∗ indicates *p* < 0.05. *F* and *G*, Western blot analysis detecting the expression levels of AR and PSA in C4-2R and 22Rv1 cells after treatment with artesunate at the indicated concentrations.

Many pieces of evidence have revealed that the AR signaling pathway plays a key role in PCa initiation and progression (20). Furthermore, the reactivation of the AR signaling pathway is closely associated with the development of ENZ-R (21, 22, 23). Thus, we wondered if ART also affects the AR signaling pathway. Analysis of RNA-seq data suggested that the AR signaling pathway was inhibited (*p* < 0.0001, NES = −2.52) following ART treatment in C4-2R cells (Fig. 4, *A* and *D*). Additionally, our qRT-PCR results showed that downstream targets of this pathway, including prostate-specific antigen (PSA), NKX3.1, TMPRSS2, and FKBP5, were significantly suppressed after ART treatment (Fig. 4*B*). Importantly, the protein level of AR decreased under ART treatment compared to the control group. In contrast, the transcription level of AR remained similar (Fig. 4, *B*, *F* and *G*). This distinction suggests that ART influences AR signaling by affecting protein stability rather than altering its transcriptional regulation. Collectively, these findings highlight the potential of ART to modulate AR signaling, contributing to its therapeutic efficacy against ENZ-R PCa cells.

### Combination of ART and ENZ exhibits synergistic effects in ENZ-R cells in vitro

**Figure 5:**
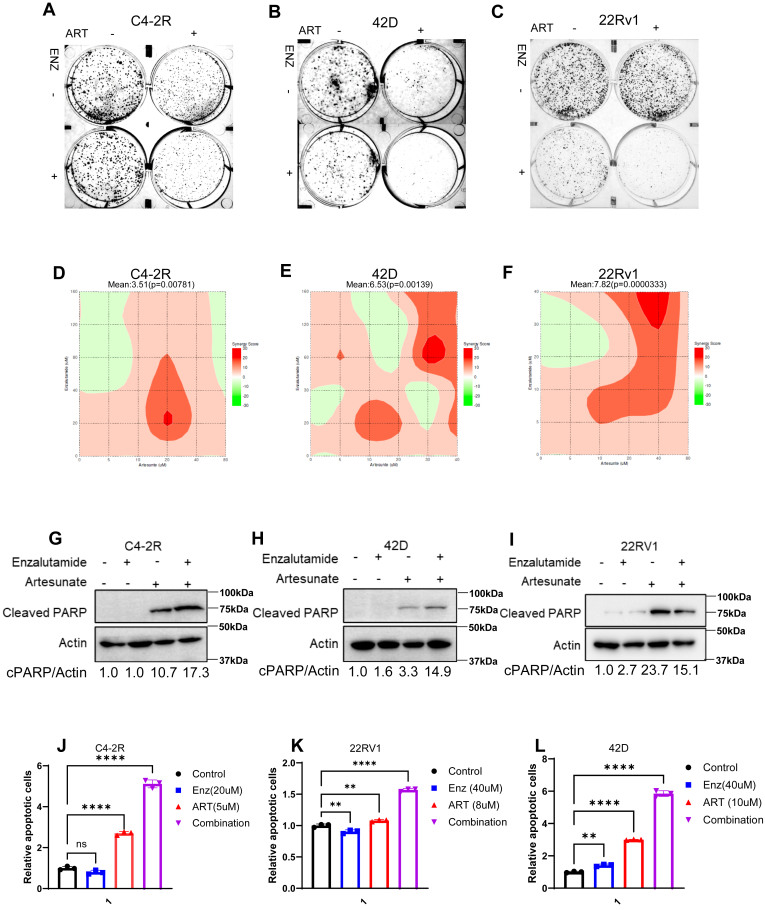
**Artesunate and enzalutamide, in combination, synergistically inhibit the growth of enzalutamide-resistant prostate cancer cells.***A*–*C*, graphic representation of the Bliss independence model of synergy scoring in C4-2R, 42D, and 22Rv1 cells. Cells were treated using a 6 × 6 grid layout with a combination of increasing concentrations of ART and ENZ for 3 days prior to assessing cell viability. *D*–*F*, 22Rv1 and C4-2R cells were cultured with ART or ENZ or combo and subjected to colony formation assay. *G*–*I*, C4-2R, 42D, and 22RV1 cells were cultured with ART or Enz for 48 h and subjected to Western blot to access cleaved PARP expression levels to evaluate the impact of these treatments on apoptosis. *J*–*L*, C4-2R, 42D, and 22RV1 cells are harvested for apoptosis analysis through flow cytometry, with results being presented as means ± S.D. of three experiments. ∗∗*p* < 0.01, ∗∗∗∗*p* < 0.0001 by the two-tailed, unpaired *t* test.

Based on the inhibitory effects of ART on c-Myc, AR, and its ability to reduce ENZ-R cell viability, we next evaluated the therapeutic potential of combining ART with ENZ *in vitro*. Given the limited benefits of ENZ monotherapy in advanced stages of PCa, we hypothesized that cotreatment with ART could enhance the anticancer effects of ENZ. Indeed, we showed that combining ART and ENZ decreases cell colony formation ability in three ENZ-R cell lines. (Fig. 5, *A*–*C*). Furthermore, combination treatment with ART and ENZ resulted in a synergistic effect in cell viability across multiple ENZ-R cell lines, including C4-2R, 22RV1, and 42D cells (Fig. 5, *D*–*F*). Bliss scores were indicated by red color intensity. A score above 10 indicates a synergistic effect, suggesting that the combination of ART and ENZ acts more effectively than mono-treatment. The combination treatment produced more significant inhibitory effects on cell viability than either drug alone. Notably, the dual therapy of ART and ENZ also led to a marked increase in apoptosis, as evidenced by higher annexin V staining (Fig. 5, *J*–*L*) and increased levels of cleaved-PARP than mono treatments (Fig. 5, *G*–*I*). Moreover, the combination therapy suppressed AR signaling more effectively than ENZ monotherapy, as indicated by a significant reduction in PSA expression, a key downstream target of AR (Fig. 3*C*). These results suggest that ART enhances the ability of ENZ to inhibit AR-driven pathways and induce apoptosis in ENZ-R PCa cells, thus providing a strong rationale for using ART in combination with ENZ to improve therapeutic outcomes in patients with drug-resistant PCa.

### ART induces c-myc degradation in a proteasomal-dependent manner

**Figure 6:**
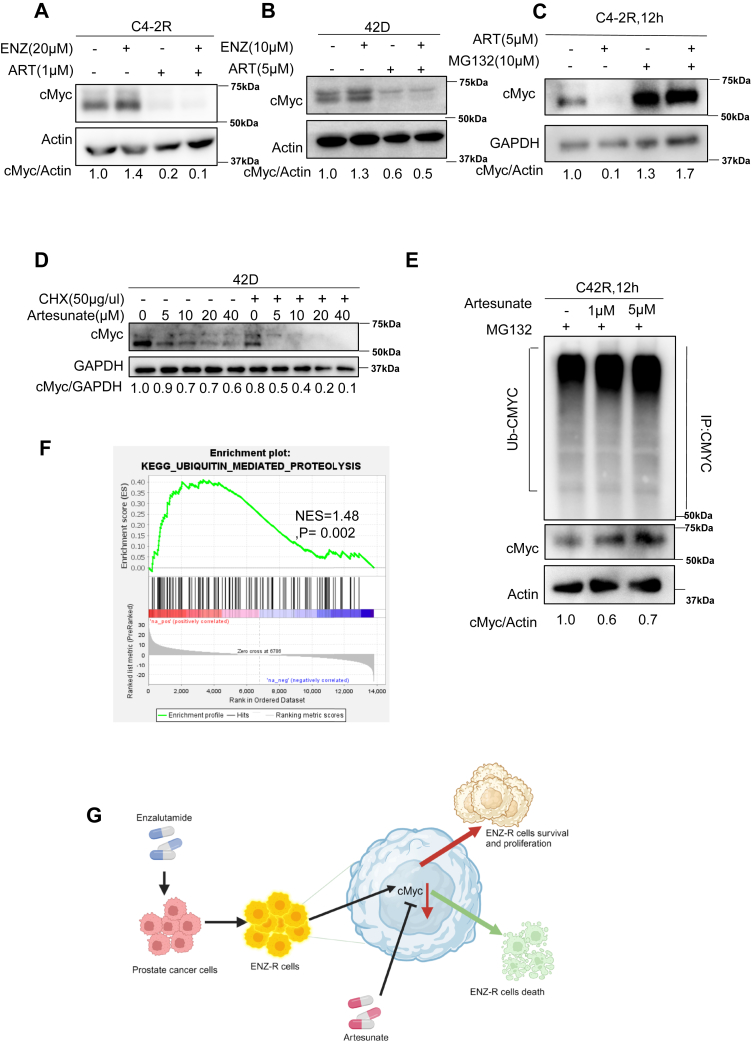
**Treatment of artesunate induces cMyc degradation in a proteasomal-dependent manner.***A* and *B*, C4-2R and 22Rv1 cells were treated with the indicated concentrations of ART and ENZ for 24 h and harvested for Western blot to measure the levels of cMyc. *C* and *E*, C4-2R cells were treated with ART for 4, followed by MG132 for 8 h. The harvested cells were subjected to Western blot or immunoprecipitation to detect c-Myc or the ubiquitination level of c-Myc. *D*, Cycloheximide (CHX) chase assay in 42D cells treated with 50 μg/mL CHX with or without artesunate (dosages indicated) over a 12-h period. Western blot analysis of c-Myc and GAPDH levels shows that artesunate accelerates c-Myc degradation over time. Quantification of c-Myc protein levels relative to GAPDH. *F*, GSEA analysis of ubiquitin-mediated proteolysis signaling from Kyoto Encyclopedia of Genes and Genomes (KEGG) gene set. *G*, working model for ART induces c-Myc degradation to target enzalutamide-resistant prostate cancer. *G*, the working model for ART in prostate cancer treatment. ART induces the proteasome degradation of c-Myc, eventually contributing to the suppression of prostate cancer cells. Since cMyc plays a significant role in developing Enz-R, combining ENZ and ART could act synergistically by suppressing c-Myc and AR signaling.

At the molecular level, we sought to explore the mechanisms through which ART exerts its antitumor effects. One key mechanism identified was the degradation of c-Myc protein. We detected decreased c-Myc protein levels across multiple ENZ-R cell lines, including C4-2R and 42D cells. This suggests that ART’s ability to reduce c-Myc protein levels is central to its anticancer activity (Fig 6, *A* and *B*). Furthermore, ART treatment significantly increased the levels of ubiquitinated c-Myc (Fig. 6*E*). Additionally, we proved that its degradation was in proteasome-dependent method (Fig. 6*C*). Meanwhile, our gene set enrichment analysis in the Kyoto Encyclopedia of Genes and Genomes dataset also showed enriched ubiquitin-mediated proteolysis (Fig. 6*F*).

c-Myc is frequently overexpressed in human cancers and regulates multiple cellular processes in cancer cells, including EMT and metabolism (24, 25). Our previous results revealed that EMT characteristics were particularly elevated in ENZ-R cells (26). Besides promoting c-Myc degradation, dual treatment with ART and ENZ inhibited ENZ-R cell migration (Fig. S1, *A*, *B* and *D*). To validate that ART induces the proteasome degradation of c-Myc in PCa, we pretreated 42D cells with cycloheximide (CHX) to inhibit protein synthesis before incubation with ART. We showed that the presence of CHX potentiated ART-associated degradation of c-Myc (Fig. 2*A*). In addition, we also pretreated C4-2R cells with ART for 3 h and added CHX. Finally, we collect cells at different time points. The result (Fig. S4*A*) is consistent with our previous finding. Futhermore, our findings also demonstrate that ART enhances the binding of FBXW7 to c-Myc (Fig. S4*B*), suggesting a potential mechanism by which ART promotes c-Myc degradation. Molecular docking studies further support this interaction, with ART showing strong binding affinity to FBXW7 in different structural models (Fig. S4, *C* and *D*). Specifically, in AlphaFold-modeled FBXW7, the binding affinity energy was calculated as −7.22 kcal/mol, whereas in the 2OVR crystal structure, it was −6.71 kcal/mol, indicating a favorable interaction. These results suggest that ART may stabilize the FBXW7–c-Myc complex, potentially facilitating c-Myc ubiquitination and degradation, thereby contributing to its anticancer effects. We further investigated the effect of ART treatment, which reduced the protein expression of mesenchymal markers such as Slug and Snail (Fig. S1*C*). Regarding metabolism, our Seahorse analysis indicated that ART inhibits oxidative phosphorylation, a key metabolic pathway supporting proliferating cancer cells' energy needs. The Seahorse assays revealed that ART treatment significantly reduced mitochondrial respiration and ATP production in ENZ-R cells. Importantly, this inhibition of oxidative phosphorylation was more pronounced when ART was combined with ENZ (Fig. S1, *F*–*I*). Thus, by inducing c-Myc degradation, a series of consequences were observed: the combination of ART and ENZ limited both the metastatic potential and energy production in ENZ-R PCa cells.

### Combination of ART and ENZ inhibits tumor growth in ENZ-R xenograft and PDX models

**Figure 7:**
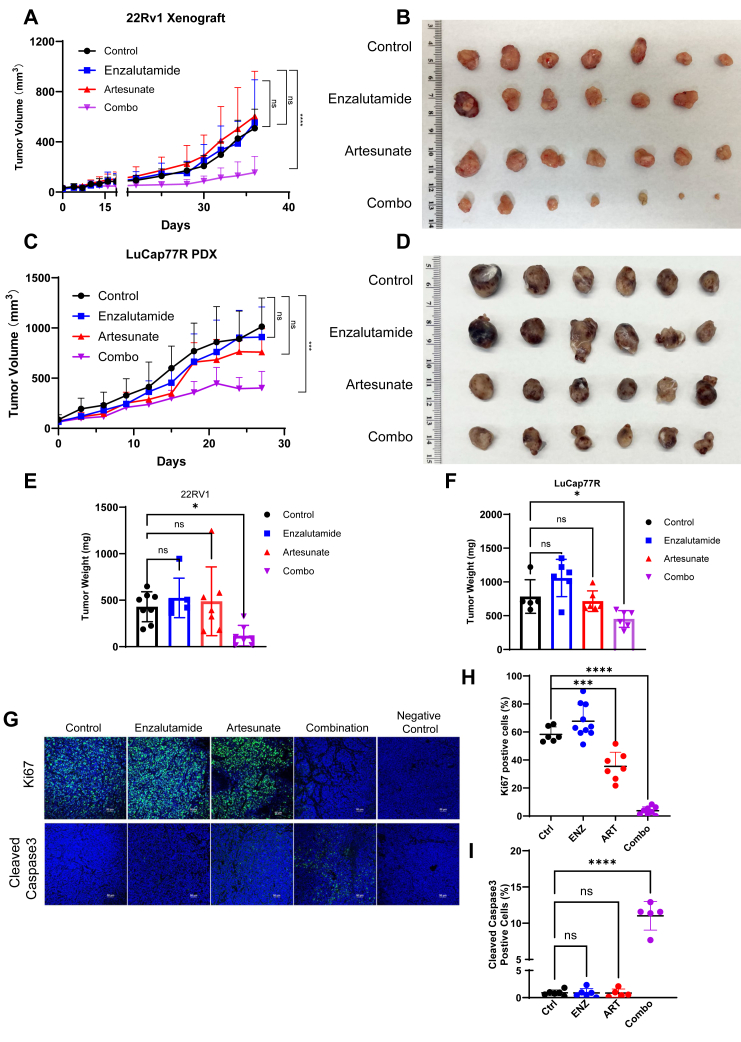
**Combination of artesunate and enzalutamide induces apoptosis *in vivo*.***A*–*D*, mice bearing 22RV1 and LuCap77CR tumors were treated with enzalutamide (ENZ, 20 mg/kg daily), artesunate (ART, 30 mg/kg daily), or a combination of both drugs (Combo). Tumor volumes were measured every 3 days, and data were presented as mean ± SD for each group (n = 7 or 6 mice per group). The combination treatment resulted in the lowest tumor growth rate. *E* and *F*, 22Rv1 and LuCap77CR tumor weight were measured after mice harvesting. The combination group showed the lowest tumor weights. ∗*p* < 0.05, ∗∗*p* < 0.01, ∗∗∗*p* < 0.001 by the two-tailed, unpaired Student’s *t* test. *G*, representative images of anti-Ki67 and anticleaved caspase3 immunofluorescent chemistry staining of 22RV1 tumor sections. Mouse IgG was used as the negative control. The results of (*H*) and (*I*) quantification of Ki67 and cleaved caspase3 signals of tumor slides are mean ± SD from four group mice. The combination treatment resulted in the lowest proliferation maker and the highest apoptosis maker expression ∗*p* < 0.05, ∗∗∗*p* < 0.001, ∗∗∗∗*p* < 0.0001 by the two-tailed, unpaired Student’s *t* test.

To determine whether the synergistic effects can be observed *in vivo*, we evaluated the combination of ART and ENZ in ENZ-R PCa xenograft models. Using both the 22RV1 xenograft model and the LuCap77R patient-derived xenograft (PDX) model, we observed a significant reduction in tumor growth in mice treated with the combination therapy compared to those treated with either ART or ENZ alone (Fig. 7, *A* and *C*). In the 22RV1 xenograft model, combination treatment with ART and ENZ resulted in a marked decrease in tumor volume compared to the control group (Fig. 7, *B* and *E*). Similar results were observed in the LuCap77R PDX model, where combination therapy significantly reduced both tumor volume and tumor weight (Fig. 7, *D* and *F*).

Moreover, immunofluorescence staining showed a substantial decrease in the proliferation marker Ki-67 in the combination treatment group compared to the monotherapy groups. Furthermore, increased expression of apoptotic markers—cleaved caspase-3—was detected in the combination group (Figs. 7, *G*–*I* and S2*A*). The combination therapy effectively inhibited proliferation and induced apoptosis in ENZ-R tumors. These *in vivo* experiments demonstrate the enhanced therapeutic potential of ART in combination with ENZ. More significantly, immunohistochemistry (IHC) staining of c-Myc showed a greatly decreased signal in both ART and combo groups, consistent with what we observed in cultured cells (Fig. S2*B*). In closing, our *in vitro* and *in vivo* experiments have fully demonstrated that ART could combine with Enz to treat Enz-R PCa. Through inducing c-Myc degradation, this novel combination therapy could significantly induce cell apoptosis and suppress cell proliferation and may further benefit clinic PCa patients.

## Discussion

In this study, we identified that ART is a potent therapeutic compound for PCa patients that enhances the efficacy of ENZ. Furthermore, ART inhibits the c-Myc and AR signaling pathway. Consequently, suppression of c-Myc–targeted cellular events, such as EMT and disruption of oxidative phosphorylation, was also detected. Mechanically, ART administration induces c-Myc degradation. In addition, our findings also revealed that ART enhances the binding of FBXW7 to c-Myc (Fig. S4*B*), suggesting a possible mechanism through which ART promotes FBXW7-mediated c-Myc degradation. Molecular docking analysis supports this interaction, demonstrating a strong binding affinity between ART and FBXW7 across different structural models (Fig. S4, *C* and *D*). Specifically, in the AlphaFold-predicted FBXW7 structure, the binding affinity energy was calculated as −7.22 kcal/mol. These results suggest that ART may cause more FBXW7 binding to c-Myc, thereby promoting c-Myc ubiquitination and degradation, contributing to its potential anticancer effects. However, further experimental validation is crucial, as computational predictions may not always accurately reflect true binding affinities.

Furthermore, the combination of ART and ENZ exhibited synergistic effects both *in vitro* and *in vivo*. Significantly enhancing the antitumor efficacy compared to monotherapy, our findings highlight the potential of ART as a novel therapeutic strategy for ENZ-ENZ-R PCa.

Given the pivotal role of c-Myc in driving PCa proliferation and resistance to AR-targeted therapies (27, 28), inhibiting c-Myc is one of the most favorable targets in ENZ-R PCa. However, plenty of clinical trials fail by directly targeting c-Myc. Recently, multiple studies discovered novel small molecules that c-Myc showed promising results (29, 30). Having a novel medication approved by the FDA is a long journey. Thus, finding beneficial medication for ENZ-R PCa patients is still challenging. Therefore, we tested old, FDA-approved drugs to get novel combination strategies. Our results show that ART exerts its anticancer effects (Fig. 1, *A*–*C*) by suppressing the c-Myc signaling pathway (Fig. 2, *A*–*E*). These findings also correlate with previous studies that have identified c-Myc as a key regulator affecting patients' response to ENZ (Fig. 3, *B*–*E*).

The synergistic effect of ART and ENZ is likely due to their complementary mechanisms of action. ENZ primarily inhibits AR signaling by disrupting androgen binding to AR (31). While c-Myc antagonist AR transcription activities (27), as a result, c-Myc elevation causes PCa to be less sensitive to ENZ treatment. Thus, targeting c-Myc restores the AR program and sensitizes PCa to ENZ. Another recent scientific publication revealed that ART could influence androgen synthesis, which contributes to reduced AR signaling in PCa cell lines (32). However, ART only partially decreases CYP11A1, an enzyme crucial for androgen production, indicating that it cannot completely halt the AR signaling pathway on its own. In contrast, ENZ is specifically designed to target AR signaling. When PCa cells develop resistance to ENZ, combining it with ART offers a comprehensive suppression of both AR signaling and other activated pathways in ENZ-R PCa cells, such as c-Myc. Thus, this combination results in synergistic effects, enhancing the therapeutic efficacy against ENZ-R PCa. Our results indicated that targeting AR and c-Myc effectively shut down two key survival pathways in PCa cells (Fig. 5, *B* and *D*). This dual action not only enhances cell death but also reduces the likelihood of resistance development, as cancer cells can compensate less for the loss of both pathways. The *in vivo* xenograft and PDX models further support the clinical relevance of this combination therapy. In both the 22RV1 xenograft and LuCap77CR PDX models, the combination of ART and ENZ resulted in significant tumor growth inhibition (Fig. 7, *A*–*F*) and increased apoptosis, as indicated by elevated levels of cleaved caspase-3 and reduced Ki-67 expression (Fig. 7*G*). Moreover, c-Myc–targeted cellular events are also suppressed by ART treatment. We detected lower cell migration ability, lower respiration ability, and ATP productivity after ART application (Fig. S1). These results provide compelling evidence that ART can enhance the therapeutic effects of ENZ in resistant PCa, offering a potential new treatment strategy for patients with limited response to current therapies.

Given ART’s favorable safety profile as an antimalarial drug (33), its repurposing for cancer therapy could provide a cost-effective and widely accessible treatment option for PCa patients. Future clinical trials are warranted to evaluate the efficacy of ART in combination with ENZ in patients with CRPC. Additionally, further research is needed to explore the molecular mechanisms underlying ART’s anticancer effects and to identify potential biomarkers that could predict response to ART treatment.

Although we identified that ART inhibits c-Myc signaling, suppresses EMT, and disrupts oxidative phosphorylation, the precise molecular mechanisms underlying these effects remain to be fully elucidated. Specifically, the detailed pathways through which ART induces c-Myc degradation and its interactions with the AR signaling axis are not entirely clear. Additional mechanistic studies, such as transcriptomic or proteomic analyses, could provide a deeper understanding of ART’s action. Regarding tumor heterogeneity and the tumor microenvironment, while we utilized xenograft and PDX models to demonstrate the efficacy of ART in combination with ENZ, these models may not fully represent the complexity of human PCa. More sophisticated models could provide more clinically relevant insights, including genetically engineered mouse models, humanized mouse models, or organoid systems derived from patients.

## Experimental procedures

### Cell culture, chemicals, and reagents

22Rv1 and 293T cells were purchased from ATCC, 42D were generously provided by Dr Amina Zoubeidi at the Vancouver Prostate Cancer Center, and the C4-2R cell line was a gift from Dr Allen Gao at the University of California, Davis. The human prostate cell lines were cultured in RPMI 1640 medium with 10% (v/v) fetal bovine serum, 100 Units/ml penicillin, and 10 μg/ml streptomycin at 37 °C in a 5% CO2 environment. The 293T cells were maintained in Dulbecco's Modified Eagle's Medium with the same fetal bovine serum and antibiotic supplementation. ART and ENZ were purchased from Medchem Express.

### Antibodies

Antibodies against AR (5153), GAPDH (5174), cleaved-caspase 3 (9661), Ki67 (9027), PSA (5365), actin (3700), c-Myc (5605) were purchased from Cell Signaling Technology. Antibody against c-Myc (MA1-980) antibodies against FLAG (F9291) was purchased from Sigma. And anti-Ub (sc-8017) antibodies were ordered from Santa Cruz Biotechnology.

### Western blot and immunoprecipitation

For cultured cells, samples were washed twice with PBS after harvesting and then digested with radio-immunoprecipitation assay lysis buffer (Sigma) supplemented with protease and phosphatase inhibitors (Sigma).

For Western blotting, proteins from each sample were mixed with SDS-PAGE loading buffer and boiled for 5 min. For immunoprecipitation, protein samples were combined with the specified antibodies and rotated at 4 °C for 4 h. Conjugated magnetic beads, as indicated in the paper (Medchem Express), were then added and incubated overnight at 4 °C. The beads were washed with Tris-buffered saline with Tween20 (TBST) following the manufacturer’s protocol, then mixed with SDS-PAGE loading buffer, and boiled for 5 min.

Following transfer to polyvinylidene difluoride membranes, proteins were probed with the indicated antibodies. Primary antibodies were diluted in 3% bovine serum albumin at a 1:1000 ratio and incubated overnight at 4 °C. The membrane was then washed three times for 5 min each with TBST and incubated with diluted secondary antibodies (1:5000) at room temperature for 1 h. Before exposure, the membrane was washed three additional times for 5 min with TBST, and signals were detected using the Clarity Western ECL Substrate (Bio-Rad). Immunoblots band signal intensity was measured by Image J and normalized by dividing the loading control intensity.

### RNA isolation, RNA-seq, and quantitative real-time PCR

After C4-2R and 22Rv1 were treated with 0.5 μM and 10 μM ART, cells were washed with cold PBS twice, followed by total RNA extraction using the RNeasy mini kit (Qiagen). For total RNA-seq, the samples were sequenced by Novogene Biotechnology Company. Six GB of data was collected for each sample. The data were analyzed for differential expression using the EdgeR R package. For qRT-PCR, extracted mRNA was reverse transcribed using the QuantiTect Reverse Transcription Kit (Qiagen), following the manufacturer’s instructions. FastStart Universal SYBR Green Master (Sigma) was employed to measure the expression levels of the target mRNA, normalized to β-actin. The qRT-PCR protocol included an initial step at 95 °C for 10 min, followed by 40 cycles of 95 °C for 15 s and 60 °C for 30 s. The primers used in the qRT-PCR are listed below.

**Table:** 

Gene	Forward	Reverse
*MYC*	GGCTCCTGGCAAAAGGTCA	CTGCGTAGTTGTGCTGATGT
*APEX1*	CAATACTGGTCAGCTCCTTCG	TGCCGTAAGAAACTTTGAGTGG
*CAD*	TGCTCACCTATCCTCTGATCG	GCTGGGAGTAGGACAGCAC
*LDHA*	AGGAGAAACACGCCTTGATTTAG	ACGAGCAGAGTCCAGATTACAA
*CDK4*	ATGGCTACCTCTCGATATGAGC	CATTGGGGACTCTCACACTCT
*HPRT1*	CCTGGCGTCGTGATTAGTGAT	AGACGTTCAGTCCTGTCCATAA
*Full-Length AR*	TCTTGTCGTCTTCGGAAATGT	AAGCCTCTCCTTCCTCCTGTA
*AR-V7*	CAGGGATGACTCTGGGAGAA	GCCCTCTAGAGCCCTCATTT
*PSA*	CAGTCTGCGGCGGTGTT	GCAAGATCACGCTTTTGTTCCT
*NKX3.1*	GGACTGAGTGAGCCTTTTGC	CAGCCAGATTTCTCCTTTGC
*TMPRSS2*	CCTCTAACTGGTGTGATGGCGT	TGCCAGGACTTCCTCTGAGATG
*CDC25C*	GAACAGGCCAAGACTGAAGC	GCCCCTGGTTAGAATCTTCCTC
*UBE2C*	GGATTTCTGCCTTCCCTGAATCA	GATAGCAGGGCGTGAGGAAC
*ACTIN*	CACCATTGGCAATGAGCGGTTC	AGGTCTTTGCGGATGTCCACGT

### Cell viability assay

Cells (2 × 10^3^ to 1 × 10^4^ per well) were seeded in 96-well plates and cultured overnight before being treated with various concentrations of drugs. After 72 h of incubation, cells were treated with Cell Titer Glo (Promega) as introduced in the manufacturing protocol. The luminescence intensity was then measured using a plate reader. IC50 values were calculated from the average viability curves generated from four independent measurements for each condition.

### Drug screening

42D cells (1 × 10^4^) were seeded in 96-well plates containing the APEXBIO drug library, consisting of 1971 FDA-approved drugs. Every concentration of drugs is 10 μM. After 72 h of incubation, cell viability was assessed by cell tier glo (Promega).

### Synergy score analysis

Drug response assays were performed using a 6 × 6 matrix design to evaluate the interactions between ART and ENZ. This approach allowed for the assessment of drug pairs both individually and in combination, utilizing five serially diluted concentrations of each drug. Cell viability was measured after a 96-h treatment using CellTiter-Glo. Each well's viability was normalized to untreated control cells grown in media with 0.2% dimethyl sulfoxide, and the percentage of viable cells was calculated. R statistical software, specifically the synergyfinder package (version 1.10.4), was employed to generate synergy scores based on the Bliss independence and Loewe additivity models.

### Colony formation assay

Cells (500–2000/well) were seeded in 6-well plates and cultured in medium alone or containing different drugs for 10 to 14 days, with the medium change every 3 days. After culturing, cells were fixed in 10% formalin and stained with 0.5% crystal violet for 30 min, followed by imaging colony.

### 22Rv1 xenograft and LuCap77CR PDX model

22Rv1 cells (2.5 × 10^6^ cells/mouse) were mixed with an equal volume of Matrigel (Corning, 356234) and subcutaneously injected into the right flank of precastrated nude mice. LuCap77CR PDX was implanted subcutaneously in NSG mice. After the tumor reached around 50 mm^3^, animals were randomized into four groups. ENZ (20 mg/kg daily) and ART (30 mg/kg daily) were dissolved in 10% DMSO and 90% corn oil and administrated through oral gavage. Tumor volumes were estimated from the formula: volume(mm^3^) = length (mm) × width(mm)^2^/2. The experiment was approved by the Institutional Animal Care and Use Committee of the University of Kentucky.

### Immunofluorescence and IHC staining

After paraffin-embedded slides were deparaffinized and rehydrated, antigen retrieval was performed using an antigen unmasking solution (Vector Laboratories, H-3301-250). The samples were then blocked and incubated with the specified primary antibodies at a 1:200 dilution, followed by incubation with secondary antibodies. For immunofluorescence staining, the slides were mounted using VECTASHIELD Antifade Mounting Medium with DAPI (Vector Laboratories, H-1200). For IHC staining, the slides were processed using the VECTASTAIN Elite ABC Universal Plus kit (Vector Laboratories, PK-8200). Representative images were captured using a Nikon microscope.

### PSA measurement

Blood was collected from mice, followed by 500 rcf centrifuge for 10 min to collect serum. As the manufacturer instructed, PSA levels were determined using a PSA ELISA kit (Sigma RAB0331).

### Apoptosis assay

Cells were detected using the APC Annexin V Apoptosis Detection Kit with 7-AAD (BioLegend 640930). The apoptosis assay was performed on a BD Symphony A2 flow cytometer, and data analysis was conducted using FlowJo software.

### Molecular docking analysis

Molecular docking simulations were conducted using the Molecular Operating Environment software to investigate the interaction between ART and FBXW7. Two structural models of FBXW7 were employed for docking: AlphaFold-predicted FBXW7 structure and crystal structure of FBXW7 (PDB ID: 2OVR).

### Statistical analysis

All numerical data are expressed as mean ± SD. Statistical significance was determined using an unpaired two-tailed Student’s *t* test, with *p* values < 0.05 considered statistically significant.

## Data availability

All data are contained within the article and supporting material.

## Supporting information

This article contains supporting information.

## Conflict of interest

The authors declare that they have no conflicts of interest with the contents of this article.
