# Supplementary material for: Artesunate enhances the efficacy of enzalutamide in advanced prostate cancer
Source: J Biol Chem. 2025 Mar 26;301(5):108458. doi: 10.1016/j.jbc.2025.108458 (PMC12051599; doi:10.1016/j.jbc.2025.108458)
Supplement: Supporting information [file mmc1.pdf]

Figure S1

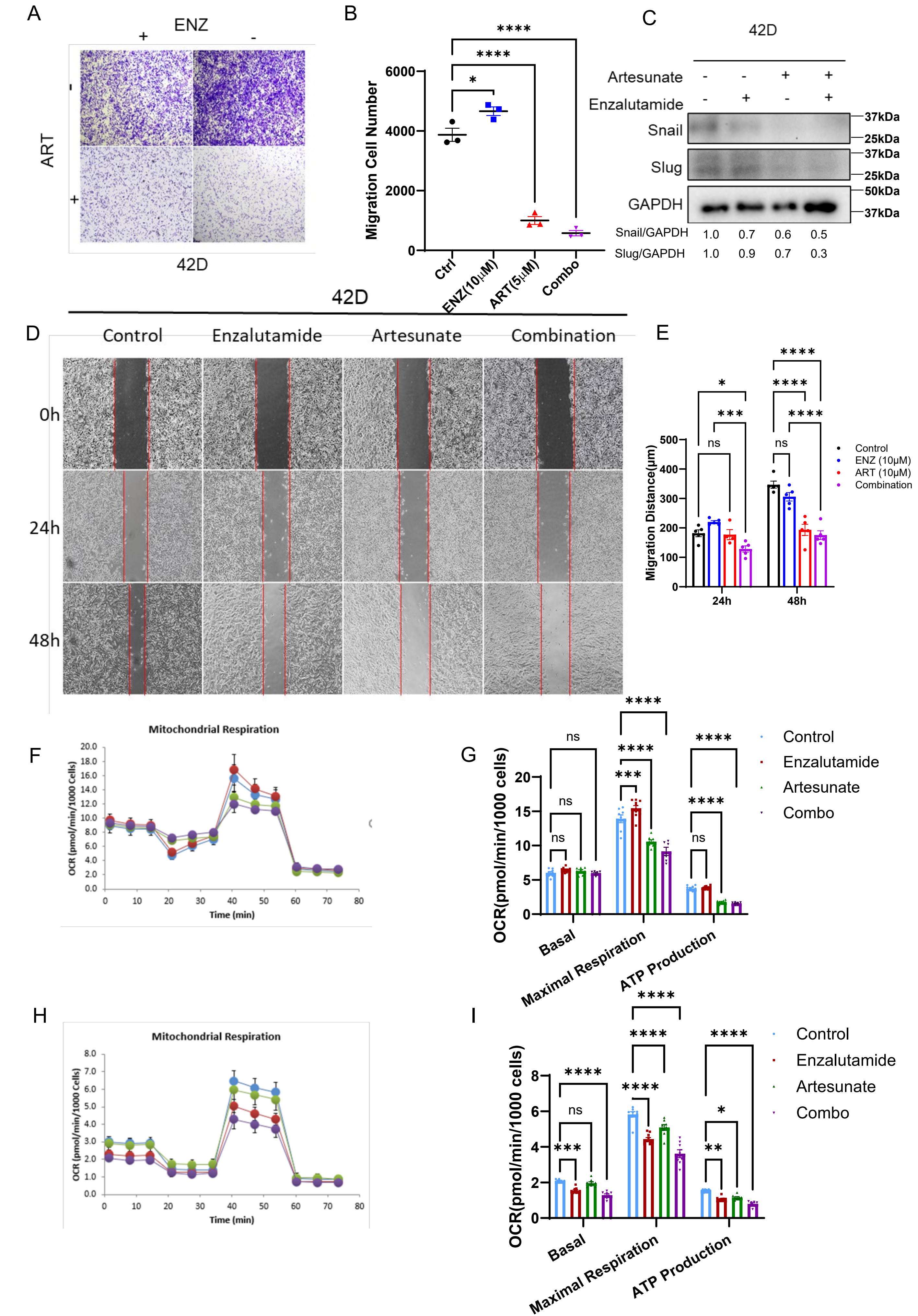

A
ENZ
+
-
ART
-
+
42D
B
Migration Cell Number
Ctrl
ENZ(10μM)
ART(5μM)
Combo
C
42D
Artesunate
Enzalutamide
Snail
Slug
GAPDH
37kDa
25kDa
50kDa
Snail/GAPDH 1.0 0.7 0.6 0.5
Slug/GAPDH 1.0 0.9 0.7 0.3
42D
D
Control
Enzalutamide
Artesunate
Combination
0h
24h
48h
E
Migration Distance(μm)
24h
48h
Control
ENZ (10μM)
ART (10μM)
Combination
F
Mitochondrial Respiration
OCR (pmol/min/1000 Cells)
Time (min)
G
OCR(pmol/min/1000 cells)
Basal
Maximal Respiration
ATP Production
Control
Enzalutamide
Artesunate
Combo
H
Mitochondrial Respiration
OCR (pmol/min/1000 Cells)
Time (min)
I
OCR(pmol/min/1000 cells)
Basal
Maximal Respiration
ATP Production
Control
Enzalutamide
Artesunate
Combo

Figure S2

A

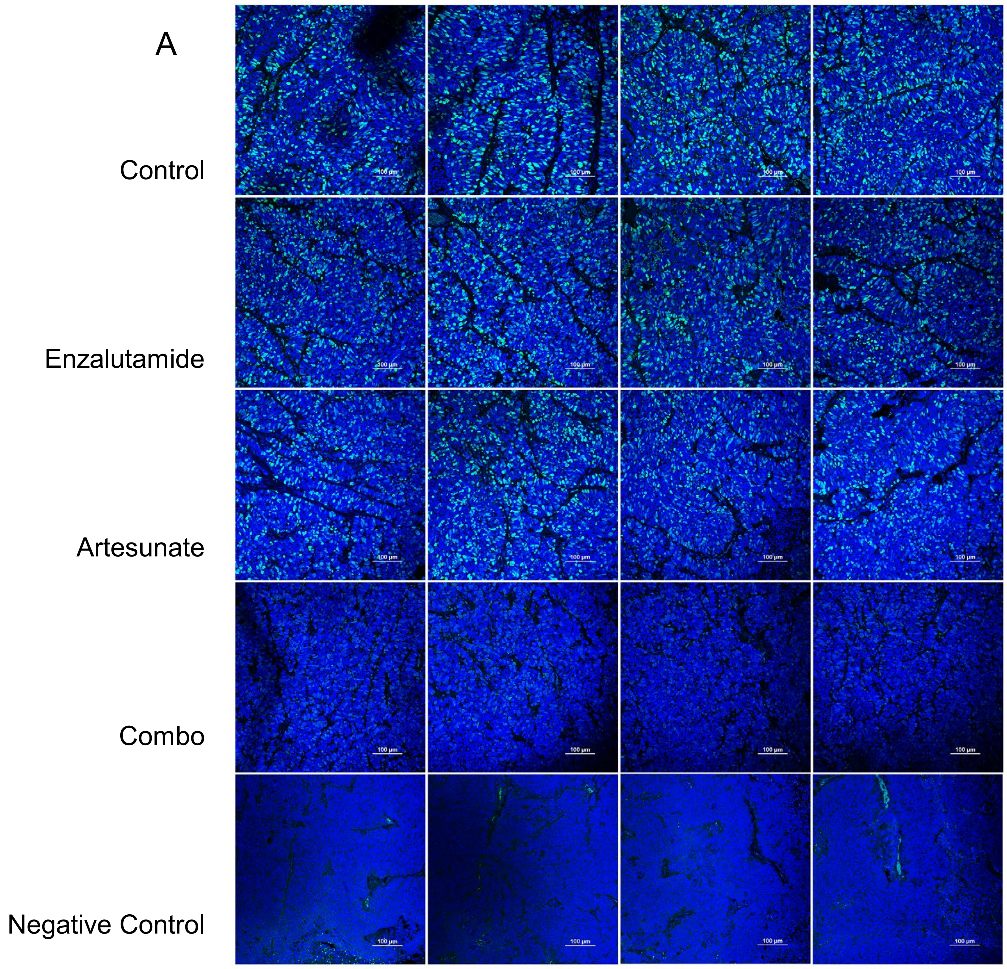
Control
Enzalutamide
Artesunate
Combo
Negative Control

B

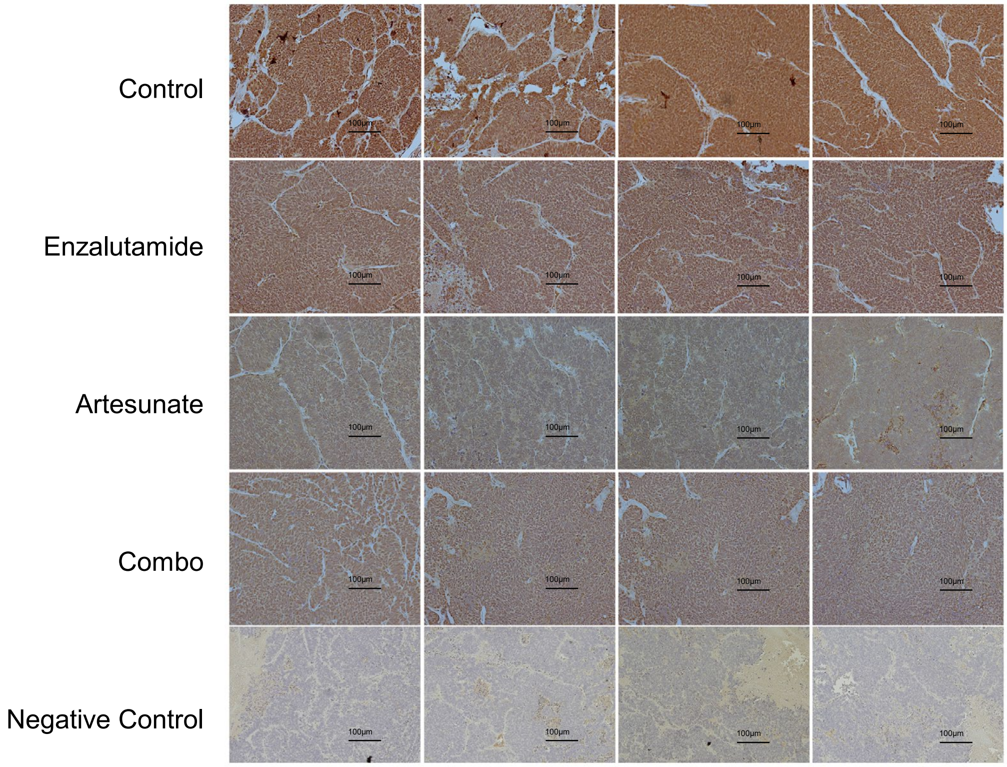
Control
Enzalutamide
Artesunate
Combo
Negative Control

Figure S3

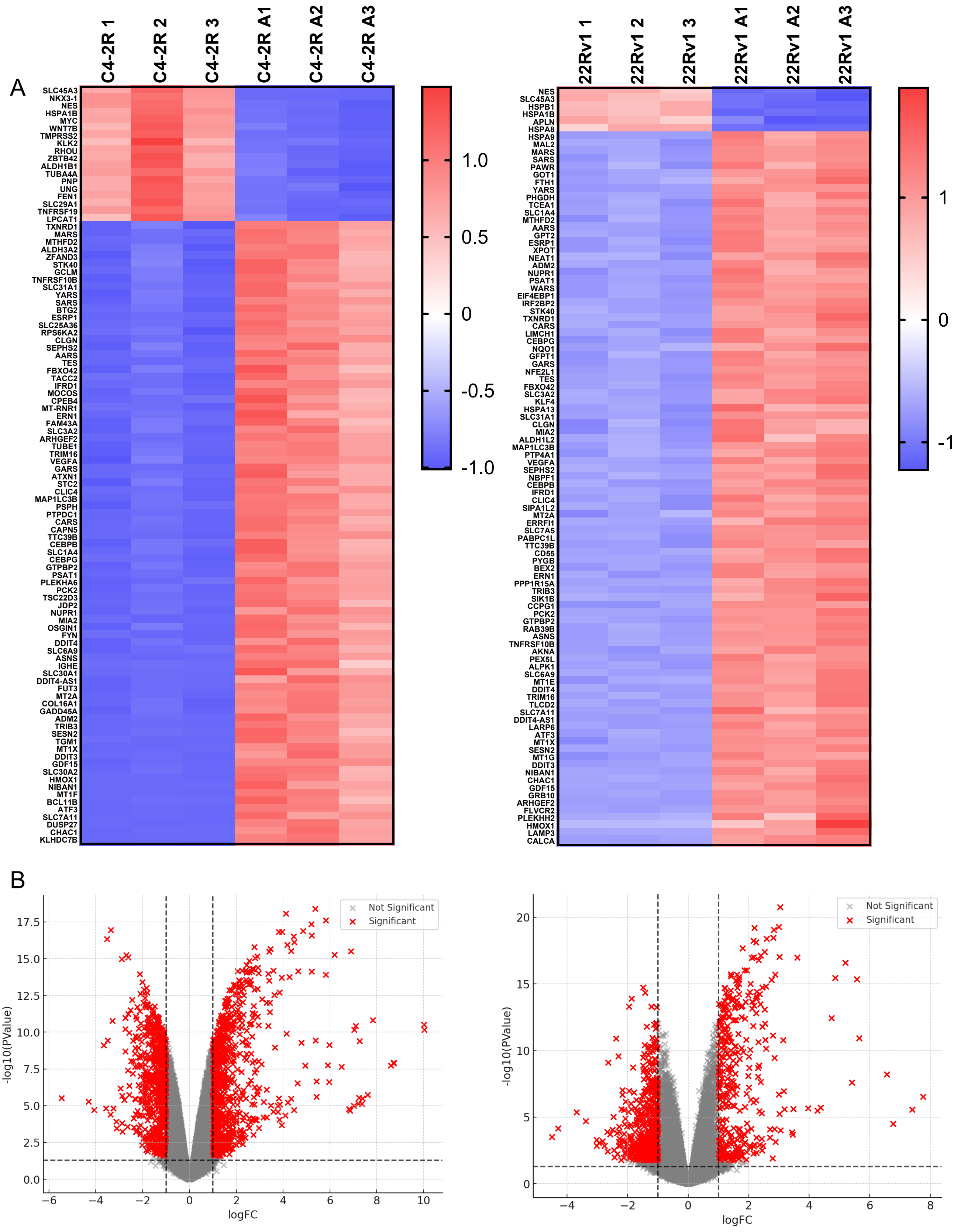
A
C4-2R 1
C4-2R 2
C4-2R 3
C4-2R A1
C4-2R A2
C4-2R A3
SLC45A3
NKX3-1
NES
HSPA1B
MYC
WNT7B
TMPRSS2
KLK2
RHOU
ZBTB42
ALDH1B1
TUBA4A
PNP
UNG
FEN1
SLC29A1
TNFRSF19
LPCAT1
TXNRD1
MARS
MTHFD2
ALDH3A2
ZFAND3
STK40
GCLM
TNFRSF10B
SLC31A1
YARS
SARS
BTG2
ESRP1
SLC25A36
RPS6KA2
CLGN
SEPHS2
AARS
TES
FBXO42
TACC2
IFRD1
MOCOS
CPEB4
MT-RNR1
ERN1
FAM43A
SLC3A2
ARHGEF2
TUBE1
TRIM16
VEGFA
GARS
ATXN1
STC2
CLIC4
MAP1LC3B
PSPH
PTPDC1
CARS
CAPN5
TTC39B
CEBPB
SLC1A4
CEBPG
GTPBP2
PSAT1
PLEKHA6
PCK2
TSC22D3
JDP2
NUPR1
MIA2
OSGIN1
FYN
DDIT4
SLC6A9
ASNS
IGHE
SLC30A1
DDIT4-AS1
FUT3
MT2A
COL16A1
GADD45A
ADM2
TRIB3
SESN2
TGM1
MT1X
DDIT3
GDF15
SLC30A2
HMOX1
NIBAN1
MT1F
BCL11B
ATF3
SLC7A11
DUSP27
CHAC1
KLHDC7B
1.0
0.5
0
-0.5
-1.0
22Rv1 1
22Rv1 2
22Rv1 3
22Rv1 A1
22Rv1 A2
22Rv1 A3
NES
SLC45A3
HSPB1
HSPA1B
APLN
HSPA8
HSPA9
MAL2
MARS
SARS
PAWR
GOT1
FTH1
YARS
PHGDH
TCEA1
SLC1A4
MTHFD2
AARS
GPT2
ESRP1
XPOT
NEAT1
ADM2
NUPR1
PSAT1
WARS
EIF4EBP1
IRF2BP2
STK40
TXNRD1
CARS
LIMCH1
CEBPG
NQO1
GFPT1
GARS
NFE2L1
TES
FBXO42
SLC3A2
KLF4
HSPA13
SLC31A1
CLGN
MIA2
ALDH1L2
MAP1LC3B
PTP4A1
VEGFA
SEPHS2
NBPF1
CEBPB
IFRD1
CLIC4
SIPA1L2
MT2A
ERRFI1
SLC7A5
PABPC1L
TTC39B
CD55
PYGB
BEX2
ERN1
PPP1R15A
TRIB3
SIK1B
CCPG1
PCK2
GTPBP2
RAB39B
ASNS
TNFRSF10B
AKNA
PEX5L
ALPK1
SLC6A9
MT1E
DDIT4
TRIM16
TLCD2
SLC7A11
DDIT4-AS1
LARP6
ATF3
MT1X
SESN2
MT1G
DDIT3
NIBAN1
CHAC1
GDF15
GRB10
ARHGEF2
FLVCR2
PLEKHH2
HMOX1
LAMP3
CALCA
1
0
-1
B
Not Significant
Significant
-log10(PValue)
logFC
0.0
2.5
5.0
7.5
10.0
12.5
15.0
17.5
−6
−4
−2
0
2
4
6
8
10
Not Significant
Significant
-log10(PValue)
logFC
0
5
10
15
20
−4
−2
0
2
4
6
8

Figure S4

A

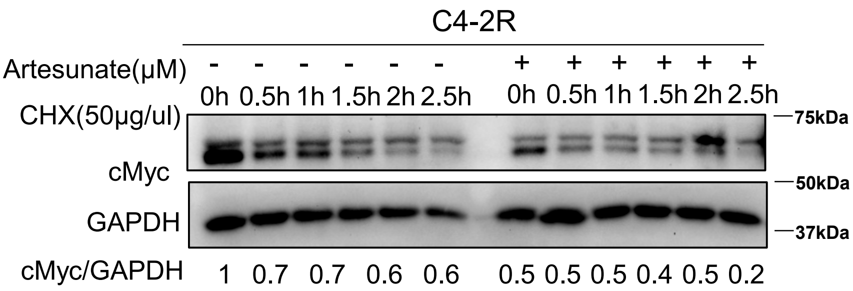

B

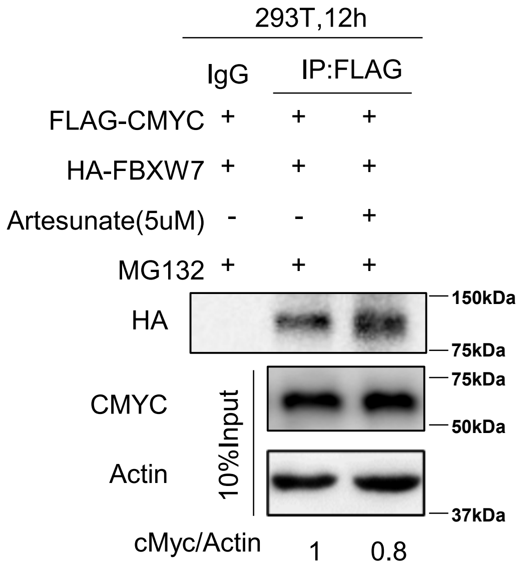

C

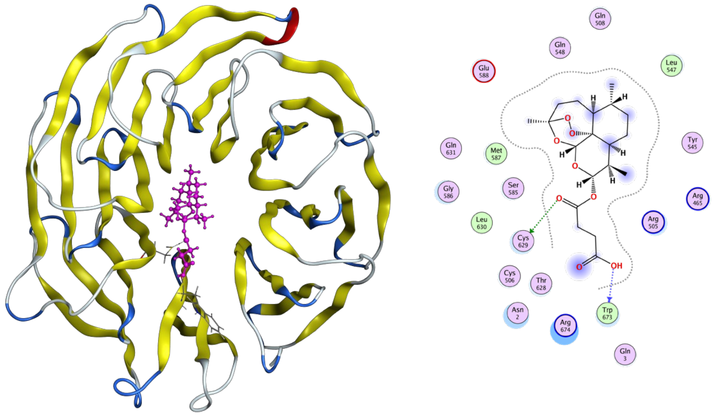

D

Protein	Structure	energy
Fbw7	AF-Q969H0-F1-v4	-7.22
	2OVR	-6.71

Figure S1. ART and ENZ combination treatment suppress EMT and Oxidative Phosphorylation.

A and **B** 48-hour in vitro migration assay using 42D cells treated with or without artesunate (ART, 10 μM) and enzalutamide (ENZ, 10 μM). The combination treatment significantly decreased cell migration ability compared to single treatments. Data are presented as mean ± standard deviation (SD) of three replicates. Statistical significance was determined using a two-tailed t-test, where * indicates $p < 0.05$, ** indicates $p < 0.01$, and *** indicates $p < 0.001$. **C,** Western blot analysis detecting EMT markers in 42D cells treated with or without ART (10 μM) and ENZ (10 μM). The combination treatment reduced the expression of EMT markers, indicating a suppression of the epithelial-to-mesenchymal transition. **D.** and **E,** wound healing assay conducted on 42D cells treated with or without ART (10 μM) and ENZ (10 μM). The combination treatment further decreased the wound closure rate, consistent with reduced cell migration ability. Data are presented as mean ± SD of three replicates. Statistical significance was determined using a two-tailed t-test, where * indicates $p < 0.05$, ** indicates $p < 0.01$, and *** indicates $p < 0.001$. F and H oxygen consumption rates (OCR) measured using a Seahorse XFe96 analyzer in C4-2R and 22RV1 cells treated with ART, ENZ, or their combination. Cells were seeded in XFe96 cell culture microplates, treated overnight, and subjected to a mitochondrial stress test. **G** and **I,** the calculated basal respiratory rate, maximal respiration, and ATP production from the OCR measurements. The data were normalized by relative cell number of treated groups compared to the control group. The combination treatment significantly decreased oxidative phosphorylation, as indicated by reduced basal and maximal respiration rates and ATP production. Data are presented as mean ±

SD of three replicates. Statistical significance was determined using a two-tailed t-test, where * indicates $p < 0.05$, ** indicates $p < 0.01$, and *** indicates $p < 0.001$, **** indicates $p < 0.0001$.

Figure S2. ART and ENZ combination treatment in vivo.

A, representative images of Ki67 immunofluorescence staining in LuCap77CR tumor slides. Ki67 is a marker of cell proliferation. The combination treatment of artesunate and enzalutamide resulted in the lowest Ki67 expression, indicating reduced tumor cell proliferation compared to single treatments. We were using a non-specific IgG of mouse as our negative control. **B,** representative images of c-Myc IHC staining in LuCap77CR tumor slides. The combination treatment showed the most significant reduction in c-Myc expression, suggesting that it effectively suppresses oncogenic pathways driving tumor growth. Mouse IgG was used as negative control.

Figure S3. ENZ-R prostate cancer cell lines induces global gene expression changes after ART treatment

A, heat map showing the top 100 gene between samples, made with the DESeq2 transformed data after a regularized log transformation was performed. **B,** volcano plots from edgeR showing, Significant genes ($FDR < 0.05$ & $|logFC| > 1$), with or without ART treatment. Dashed lines: Thresholds for significance. The x axis shows the average log (count per million, CPM), and the y axis shows the log10 (fold change).

Figure S4. Artesunate Enhances FBXW7–c-Myc Interaction and Promotes c-Myc Degradation

A, Cycloheximide (CHX) chase assay in C4-2R cells treated with 50 µg/mL CHX with or without artesunate (5 µM) over a 2.5-hour period. Western blot analysis of c-Myc and GAPDH levels shows that artesunate accelerates c-Myc degradation over time. Quantification of c-Myc protein levels relative to GAPDH. **B,** Co-immunoprecipitation assay showing the interaction between FBXW7 and c-Myc in 293T cells treated with 5 µM artesunate for 12 hours in the presence of MG132. Immunoprecipitation was performed using an anti-FLAG antibody, followed by western blot analysis for c-Myc. IgG served as a negative control. **C** and **D,** Molecular docking analysis of artesunate binding to FBXW7 using Molecular Operating Environment (MOE) software. Binding affinity calculations show a binding energy of -7.22 kcal/mol in the Alpha Fold-modeled FBXW7 structure (AF-Q969H0-F1-v4) and -6.71 kcal/mol in the 2OVR crystal structure, indicating a favorable interaction between ART and FBXW7.

Figure S1. ART and ENZ combination treatment suppress EMT and Oxidative Phosphorylation.

A and **B** 48-hour in vitro migration assay using 42D cells treated with or without artesunate (ART, 10 μM) and enzalutamide (ENZ, 10 μM). The combination treatment significantly decreased cell migration ability compared to single treatments. Data are presented as mean ± standard deviation (SD) of three replicates. Statistical significance was determined using a two-tailed t-test, where * indicates $p < 0.05$, and **** indicates $p < 0.0001$. **C,** Western blot analysis detecting EMT markers in 42D cells treated with or without ART (10 μM) and ENZ (10 μM). The combination treatment reduced the expression of EMT markers, indicating a suppression of the epithelial-to-mesenchymal transition. **D.** and **E,** wound healing assay conducted on 42D cells treated with or without ART (10 μM) and ENZ (10 μM). The combination treatment further decreased the wound closure rate, consistent with reduced cell migration ability. Data are presented as mean ± SD of three replicates. Statistical significance was determined using a two-tailed t-test, where * indicates $p < 0.05$, and *** indicates $p < 0.001$, **** indicates $p < 0.0001$. F and H oxygen consumption rates (OCR) measured using a Seahorse XFe96 analyzer in C4-2R and 22RV1 cells treated with ART, ENZ, or their combination. Cells were seeded in XFe96 cell culture microplates, treated overnight, and subjected to a mitochondrial stress test. **G** and **I,** the calculated basal respiratory rate, maximal respiration, and ATP production from the OCR measurements. The data were normalized by relative cell number of treated groups compared to the control group. The combination treatment significantly decreased oxidative phosphorylation, as indicated by reduced basal and maximal respiration rates and ATP production. Data are presented as mean ± SD of three

replicates. Statistical significance was determined using a two-tailed t-test, where *** indicates $p < 0.001$, and **** indicates $p < 0.0001$.

Figure S2. ART and ENZ combination treatment in vivo.

A, representative images of Ki67 immunofluorescence staining in LuCap77CR tumor slides. Ki67 is a marker of cell proliferation. The combination treatment of artesunate and enzalutamide resulted in the lowest Ki67 expression, indicating reduced tumor cell proliferation compared to single treatments. We were using a non-specific IgG of mouse as our negative control. **B,** representative images of c-Myc IHC staining in LuCap77CR tumor slides. The combination treatment showed the most significant reduction in c-Myc expression, suggesting that it effectively suppresses oncogenic pathways driving tumor growth. Mouse IgG was used as negative control.

Figure S3. ENZ-R prostate cancer cell lines induces global gene expression changes after ART treatment

A, heat map showing the top 100 gene between samples, made with the DESeq2 transformed data after a regularized log transformation was performed. **B,** volcano plots from edgeR showing, Significant genes ($FDR < 0.05$ & $|logFC| > 1$), with or without ART treatment. Dashed lines: Thresholds for significance. The x axis shows the average log (count per million, CPM), and the y axis shows the log10 (fold change).

Figure S4. Artesunate Enhances FBXW7–c-Myc Interaction and Promotes c-Myc Degradation

A, Cycloheximide (CHX) chase assay in C4-2R cells treated with 50 μg/mL CHX with or without artesunate (5 μM) over a 2.5-hour period. Western blot analysis of c-Myc and GAPDH levels shows that artesunate accelerates c-Myc degradation over time. Quantification of c-Myc protein levels relative to GAPDH. **B,** Co-immunoprecipitation assay showing the interaction between FBXW7 and c-Myc in 293T cells treated with 5 μM artesunate for 12 hours in the presence of MG132. Immunoprecipitation was performed using an anti-FLAG antibody, followed by western blot analysis for c-Myc. IgG served as a negative control. **C** and **D,** Molecular docking analysis of artesunate binding to FBXW7 using Molecular Operating Environment (MOE) software. Binding affinity calculations show a binding energy of -7.22 kcal/mol in the Alpha Fold-modeled FBXW7 structure (AF-Q969H0-F1-v4) and -6.71 kcal/mol in the 2OVR crystal structure, indicating a favorable interaction between ART and FBXW7.
